# An Atherogenic Diet Disturbs *Aquaporin 5* Expression in Liver and Adipocyte Tissues of Apolipoprotein E-Deficient Mice: New Insights into an Old Model of Experimental Atherosclerosis

**DOI:** 10.3390/biomedicines9020150

**Published:** 2021-02-04

**Authors:** Inês V. da Silva, Courtney A. Whalen, Floyd J. Mattie, Cristina Florindo, Neil K. Huang, Sandra G. Heil, Thomas Neuberger, A. Catharine Ross, Graça Soveral, Rita Castro

**Affiliations:** 1 Research Institute for Medicines (iMed.ULisboa), Faculty of Pharmacy, Universidade de Lisboa, 1649-003 Lisboa, Portugal; imsilva1@campus.ul.pt; 2 Department of Pharmaceutical Sciences and Medicines, Faculty of Pharmacy, Universidade de Lisboa, 1649-003 Lisboa, Portugal; cristinaflorindo@ff.ulisboa.pt; 3 Department of Nutritional Sciences, The Pennsylvania State University, University Park, PA 16802, USA; caw400@psu.edu (C.A.W.); fjm1311@gmail.com (F.J.M.); neil.huang@tufts.edu (N.K.H.); acr6@psu.edu (A.C.R.); 4 Cardiovascular Nutrition Laboratory, Jean Mayer USDA Human Nutrition Research Center on Aging, Tufts University, Boston, MA 02111, USA; 5 Department of Clinical Chemistry, Medical Center Rotterdam, Erasmus MC University, 3015 GD Rotterdam, The Netherlands; s.heil@erasmusmc.nl; 6 Huck Institutes of the Life Sciences, The Pennsylvania State University, University Park, PA 16802, USA; tun3@psu.edu; 7 Department of Biomedical Engineering, The Pennsylvania State University, University Park, PA 16802, USA

**Keywords:** MRI (magnetic resonance imaging), endothelial dysfunction, high-fat diets, plaque burden

## Abstract

The dysfunction of vascular endothelial cells is profoundly implicated in the pathogenesis of atherosclerosis and cardiovascular disease, the global leading cause of death. Aquaporins (AQPs) are membrane channels that facilitate water and glycerol transport across cellular membranes recently implicated in the homeostasis of the cardiovascular system. Apolipoprotein-E deficient (*apoE*^−/−^) mice are a common model to study the progression of atherosclerosis. Nevertheless, the pattern of expression of *AQPs* in this atheroprone model is poorly characterized. In this study, *apoE*^−/−^ mice were fed an atherogenic high-fat (HF) or a control diet. Plasma was collected at multiple time points to assess metabolic disturbances. At the endpoint, the aortic atherosclerotic burden was quantified using high field magnetic resonance imaging. Moreover, the transcriptional levels of several *AQP* isoforms were evaluated in the liver, white adipocyte tissue (WAT), and brown adipocyte tissue (BAT). The results revealed that HF-fed mice, when compared to controls, presented an exacerbated systemic inflammation and atherosclerotic phenotype, with no major differences in systemic methylation status, circulating amino acids, or plasma total glutathione. Moreover, an overexpression of the isoform *AQP5* was detected in all studied tissues from HF-fed mice when compared to controls. These results suggest a novel role for AQP5 on diet-induced atherosclerosis that warrants further investigation.

## 1. Introduction

Despite significant advances in the treatment of cardiovascular disease (CVD), it remains the leading cause of mortality and morbidity among adults [1]. The major cause of CVD is atherosclerosis that is elicited by the early impairment of endothelial function and results in a chronic inflammatory condition in which arteries harden through the build-up of lipid-rich plaque in the vessel wall [2]. 

Aquaporins (AQPs) have been recently proposed to contribute to the homeostasis of the cardiovascular system [3,4]. Aquaporins are channel-forming proteins that facilitate water and small solutes transport across the plasma membrane driven by osmotic or solute gradients [5,6]. These channels play a variety of important physiological roles in mammals [7]. In humans, the 13 isoforms (AQP0-12) are categorized according to structural and functional properties, where the orthodox aquaporins (AQP0, 1, 2, 4, 5, 6, and 8) are mainly water channels; aquaglyceroporins (AQP3, 7, 9, and 10) also transport small uncharged solutes, such as glycerol and urea; and the less well-known S-aquaporins (AQP11 and 12) are under investigation with respect to their subcellular localization and selectivity [8,9,10]. Recent evidence has shown that AQP3, 5, 8, and 9 also facilitate the permeation of the major reactive oxygen species (ROS), hydrogen peroxide, being thus termed peroxiporins [11,12,13]. As facilitators of water and glycerol membrane permeation, aquaglyceroporins are crucial for energy production in different organs [14,15]. In circumstances of negative energy balance, glycerol produced by triacylglycerol (TAG) lipolysis in the white adipose tissue (WAT) is released into the bloodstream via AQP7 and used in peripheral tissues as an energy source [16,17]. In the liver, the main organ responsible for whole-body glycerol metabolism, glycerol is taken up via AQP9 to be used for gluconeogenesis [18,19]. The insulin-dependent glucose uptake is also induced by blood glycerol concentration that, by using the AQP7 route, induces pancreatic β-cells to secrete insulin [20]. Thus, the close coordination of aquaglyceroporins in metabolic-related organs is crucial for control of whole-body energy homeostasis and lipid accumulation [15,21]. The balance between the two types of adipose depots, WAT, and brown adipose tissue (BAT) impacts energy homeostasis through their specific metabolic and endocrine functions [22,23]. Adipose tissue releases a large number of adipokines and bioactive mediators that influence not only bodyweight homeostasis but also inflammation, which is a major driver of atherosclerosis [24]. While WAT is an anabolic tissue involved in energy storage, with deleterious consequences for metabolic health, BAT is catabolic and involved in energy production in the form of heat, conferring beneficial effects on adiposity. In consequence, the browning of WAT has been described as a potential strategy to target and control obesity that is causally related to CVD [25]. Our previous work revealed that AQP7 and AQP9 are downregulated along the browning process, which may be related to the physiological role of BAT in heat production, contrasting with the anabolic/catabolic lipid metabolism in white adipose cells [26].

Consistent with the vital role of AQPs in maintaining body water and energy homeostasis, alterations in their physiological functions have been related with the development of cardiometabolic risk factors [22,23]. For instance, the functional importance of AQP1 to maintain endothelial homeostasis and cardiovascular health in humans and mice has been recently reported [4]. Moreover, our prior in vitro study confirmed endothelial AQP1 as a candidate player in the setting of endothelial dysfunction, an early hallmark of atherosclerosis and CVD [14]. Following the emergent importance of AQPs in health and disease [27], these proteins are being viewed as promising novel therapeutic targets for several disorders including the metabolic syndrome, a cluster of atherosclerotic cardiovascular disease risk factors including visceral adiposity, insulin resistance, and dyslipidemia [15].

Apolipoprotein E deficient (*apoE*^−/−^) mice have been widely used as an animal model to study the pathophysiology of atherosclerosis due to the striking similarities with humans on the molecular mechanisms that lead to endothelial dysfunction and vascular plaque formation [2,3,4,25,28,29]. Nevertheless, neither the pattern of *AQPs* expression in this atheroprone model is characterized nor the corresponding influence of disease progression. As aforementioned, the functional importance of AQPs on cardiovascular homeostasis has been recently suggested. Thus, we postulated that an atherogenic diet would disturb *AQPs* expression profile, allowing the identification of the *AQPs* isoforms related with vascular toxicity. To investigate this possibility male *apoE*^−/−^ mice were fed high-fat (HF) or control diets. We assessed systemic inflammation, other metabolic disturbances, and the extent of vascular atherosclerotic lesions formation using biochemical analyses and high-field magnetic resonance imaging (MRI). After confirming a strong atherosclerotic phenotype in the HF-fed mice, we determined the mRNA expressions of the orthodox isoforms, *AQP1* and *AQP5*, and of the aquaglyceroporins *AQP3*, *AQP7,* and *AQP9*, in different metabolic tissues (BAT, WAT, and liver). 

## 2. Experimental Section

### 2.1. Animals and Diets 

Seven-week-old male *apoE*^−/−^ mice (Jackson Laboratory, Bar Harbor, ME, USA) were fed one of the following diets prepared based on AIN 93M recommendations but with different levels of fat/cholesterol (Research Diets, New Brunswick, NJ, USA). A control diet (C, 5% *w*/*w* fat) or a High Fat diet (HF, 20% *w*/*w* fat and 0.15% cholesterol). Macronutrient dietary composition is shown in Table A1. Animals were housed in a room at 22 ± 2 °C with a 12-h light-dark cycle with free access to food and water during 14 (±2) weeks. Diets were refreshed weekly, at which time animals and their remaining food were weighed. Food consumption was estimated as an average per mouse per day within each cage. All procedures were performed in compliance with the Institutional Animal Care and Use Committee of the Pennsylvania State University, which specifically approved this study.

### 2.2. Blood Collection 

At different time points, blood was collected from the retroorbital sinus of anesthetized animals into heparinized tubes and immediately put on ice. Within 30 min, plasma was isolated by centrifugation at 2000 rpm, 15 min, at 4 °C and stored at −80 °C prior to further biochemical analyses. Due to the limited volume of blood obtained, samples obtained at different time-points were used in the subsequent biochemical analysis, as further detailed.

### 2.3. Biochemical Analyses

#### 2.3.1. Systemic Methylation Index

The effect of 8 weeks of experimental diets on systemic methylation index was evaluated by the ratio of the plasmatic concentrations of S-adenosylmethionine (AdoMet) to S-adenosylhomocysteine (AdoHcy) (AdoMet/AdoHcy ratio) quantified by liquid chromatography tandem-mass spectrometry (LC-MS/MS), as previously described [30].

#### 2.3.2. Triacylglycerols 

The effect of 10 weeks of experimental diets on the fasting plasma concentrations of TAG were measured using a colorimetric assay (Cayman, Ann Arbor, MI, USA) following the manufacturer’s protocol.

#### 2.3.3. Cytokines

The effect of 12 weeks of experimental diets on the plasma concentrations of several proinflammatory cytokines/adipokines (interleukin 6, IL-6; macrophage inflammatory protein-1 alpha, MIP-1α; monocyte chemoattractant protein 1, MCP-1; and tumor necrosis factor α, TNF-α) were determined using MSD U-PLEX multiplex assay platforms (Meso Scale Diagnostics, Rockville, MD, USA) following the manufacturer’s instructions. 

#### 2.3.4. Plasma Amino Acids and Glutathione

Plasma concentrations of amino acids and total glutathione after 12 weeks on each diet were quantified by adequate methodology. Specifically, amino acids were determined by gas chromatography-flame ionization detector (GC-FID) using the Phenomenex EZ:faastTM kit for physiological amino acid analysis (Phenomenex. Torrance, CA, USA) according to the manufacturer’s instructions and as previously described in detail [31]. Total glutathione, defined as the total concentration of glutathione after reductive cleavage of all disulfide bonds, was quantified by high-performance liquid chromatography (HPLC) analysis with fluorometric detection, as previously described [32]. 

#### 2.3.5. Glucose 

At the end of each experiment, fasting blood glucose levels were determined using a glucometer (Contour, Bayer, Tarrytown, NY, USA) following the manufacturer’s instructions. 

### 2.4. Tissue Collection

At the end-point mice were euthanized by carbon dioxide inhalation and aortas were collected as previously described in detail [33]. Inguinal subcutaneous WAT and interscapular BAT were removed and immediately snap-frozen in liquid nitrogen. Livers were removed, weighted, sampled, and embedded in Optimal Cutting Temperature (OCT) compound (Sakura Finetek, Torrance, CA, USA) or immediately snap-frozen in liquid nitrogen. All samples were stored at −80 °C before further analyses. 

### 2.5. Oil Red O Staining

Oil Red *O* staining was used to determine the distribution of lipid droplets in the liver. The OCT-frozen samples were cut into 8-µm-thick sections, fixed in 4% formaldehyde (Thermo Fisher Scientific, Waltham, MA, USA) for 10 min, and rinsed in deionized water and dried at room temperature before Oil Red O staining (Sigma-Aldrich, St. Louis, MO). The sections were stained for 20 min with Oil Red O and rinsed in tap water for 5 min. Hematoxylin (Sigma-Aldrich, St. Louis, MO, USA) was counterstained to assist tissue visualization, and the slides were stained for 1 min and rinsed in tap water for 5 min. All of the representative images are at 400X magnification. The detailed information was described previously [34]. 

### 2.6. RNA Extraction 

Total RNAs from WAT, BAT, and liver were extracted using the Qiagen RNeasy lipid tissue mini kit and Qiagen RNeasy mini kit, respectively (Qiagen, Germantown, MD, USA) and were stored at −80 °C prior to analysis. To exclude possible DNA contamination, the optional on-column DNA digestion with the RNase-free DNase (Qiagen) was performed during RNA extraction. All procedures were conducted by following the manufacturer’s protocol. The RNA concentrations were determined spectrophotometrically at 260 nm using the NanoDrop 2000c (ThermoFisher Scientific, Waltham, MA, USA). The 260/280 nm ratio was utilized to assess the purity of RNA samples. Only samples with 260/280 nm ratios between 1.8 and 2.2 were used for cDNA synthesis. Additionally, agarose bleach gels were used to qualitatively assess RNA integrity by visualization of the 28S and 18S rRNA bands [35]. To generate cDNA for quantitative PCR, 500 ng of total RNA was reverse-transcribed for 1 h at 42 °C using M-MLV reverse transcriptase (Promega, Madison, WI, USA) and oligo(dT)15 Primer (Promega, Madison, WI, USA).

### 2.7. Quantitative PCR Analysis

RNA samples were reverse transcribed using M-MLV reverse transcriptase (Promega, Madison, WI, USA) with oligo dT primers (Promega, Madison, WI, USA). Real-time PCR reactions were carried out using a CFX96 Real-Time System C1000 (BioRad, Hercules, CA, USA), the TaqMan Universal PCR Master Mix (Applied Biosystems, Foster City, CA, USA) and the following specific TaqMan pre-designed gene expression primers and probes (Applied Biosystems, Foster City, CA, USA: AQP1 (#Mm00431834_m1), AQP3 (#Mm01208559_m1), AQP5 (#Mm00437578_m1), AQP7 (#Mm00431839_m10), AQP9 (#Mm00508094_m1), and Eef2 (#Mm00833287_g1) as previously described in [26]. The Ct method (2-ΔΔCt) was used for relative quantification of target genes expression after normalization with the Eef2 reference gene [36,37].

### 2.8. Quantification of Aortic Plaque Volume

Aortas were processed as previously described in detail [33]. Briefly, dissected aortas were equilibrated to 0.1% Magnevist (Bayer HealthCare Pharmaceuticals Inc., Whippany, NJ, USA), 0.25% sodium azide, PBS solution overnight at 4 °C, and plaque volume was determined by MRI using an Agilent 14T micro imaging system (Agilent Technologies, Inc., Santa Clara, CA, USA). After acquisition MR data was reconstructed using Matlab (The Mathworks Inc., Natick, MA, USA) and data segmentation was performed using Avizo 9.5 (Themo Fisher Scientific, Waltham, MA, USA). The lumen of the aorta, the different plaques, and the aorta wall were manually segmented. Quantification of plaque volume was obtained using the material statistics function on the segmented aorta and the results were expressed as the % of plaque volume in relation to the total segmented volume.

### 2.9. Statistical Analysis

Statistical analyses were performed in GraphPad Prism 7 (GraphPad Software, La Jolla, CA, USA), with statistical significance set to *p* < 0.05. For two-group comparison, an unpaired Student’s *t*-test was used. For more than two groups, a one-analysis of variance (ANOVA) was performed, followed by Tukey’s.

## 3. Results and Discussion

To investigate whether a HF diet would disturb the expression of AQPs in an atherosclerosis prone model, we fed *apoE*^−/−^ mice HF or control diets. Only male mice were included to control for the known effect of gender on atherosclerosis in this strain [38].

### 3.1. Diet Consumption, Body and Liver Weights, and Liver Histology 

**Figure 1:**
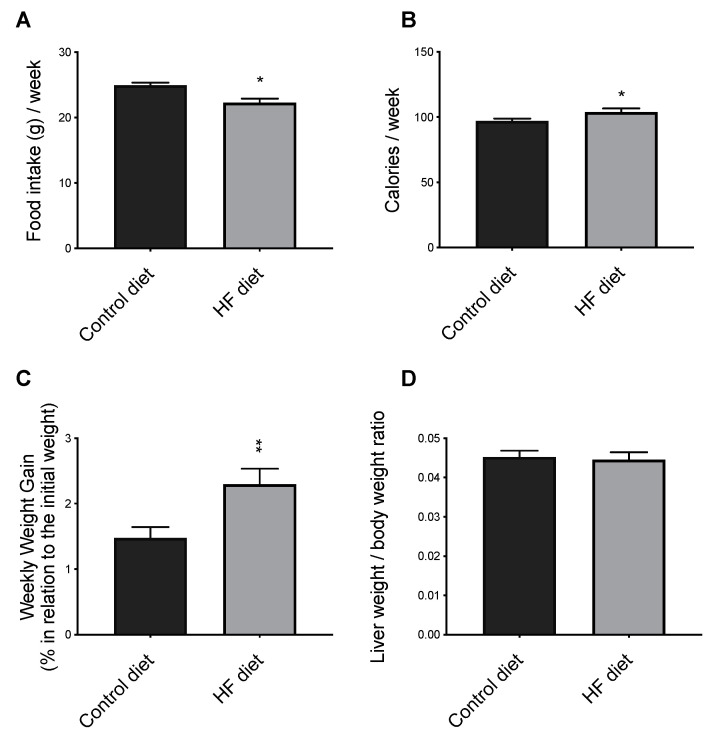
The effect of control and High-Fat (HF) diets on food (**A**) and calories (**B**) intake, and on animal growth (**C**) and relative liver weights (**D**). Data shown are the mean ± SEM (n = 10 per group); * *p* < 0.05; ** *p* < 0.01 control versus HF.

The results showed that HF mice consumed significantly less food than the mice fed a control diet (Figure 1A), nevertheless due to the higher energy density of the HF diet, more calories were consumed by HF-fed mice versus controls (Figure 1B). As expected, HF mice gained more weight compared to controls [39,40] (Figure 1C). No differences were observed in absolute liver weights (g) between the two groups of mice. The values (mean ± SEM, n = 10/group) were 1.17 ± 0.09 and 1.22 ± 0.13, for controls and HF, respectively. The relative liver weights were also similar in the two groups (Figure 1D). 

**Figure 2:**
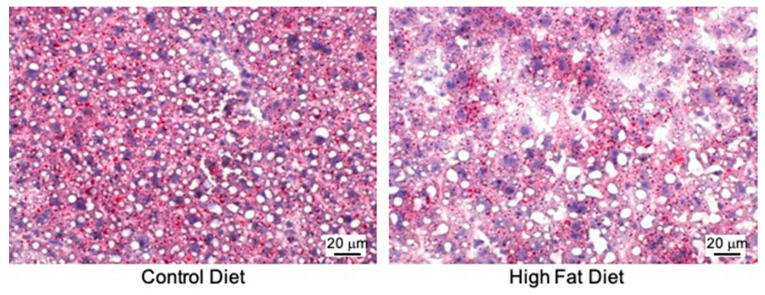
The effect of the experimental diets on liver morphology and hepatic lipid deposition (n = 3–4/group). The images selected as representatives are at 400× magnification. Red: neutral lipid; purple: nuclei.

Oil Red *O* staining showed that livers from HF-fed mice presented more macrovesicular lipid droplets of Oil Red *O* positive staining than controls (Figure 2). Ballooned hepatocytes and sinusoids capillarization, on the other hand, were also exacerbated in the HF-fed mice, showing more inflated hepatocytes, as compared to the control mice, indicating liver damage by the HF diet. This finding is consistent with a previous study, in which hepatic fat accumulation was reported in *apoE*^−/−^ mice after seven weeks of a diet containing similar amounts of fat and cholesterol as the HF-diet used in this study [41].

### 3.2. Blood Biochemistry

**Figure 3:**
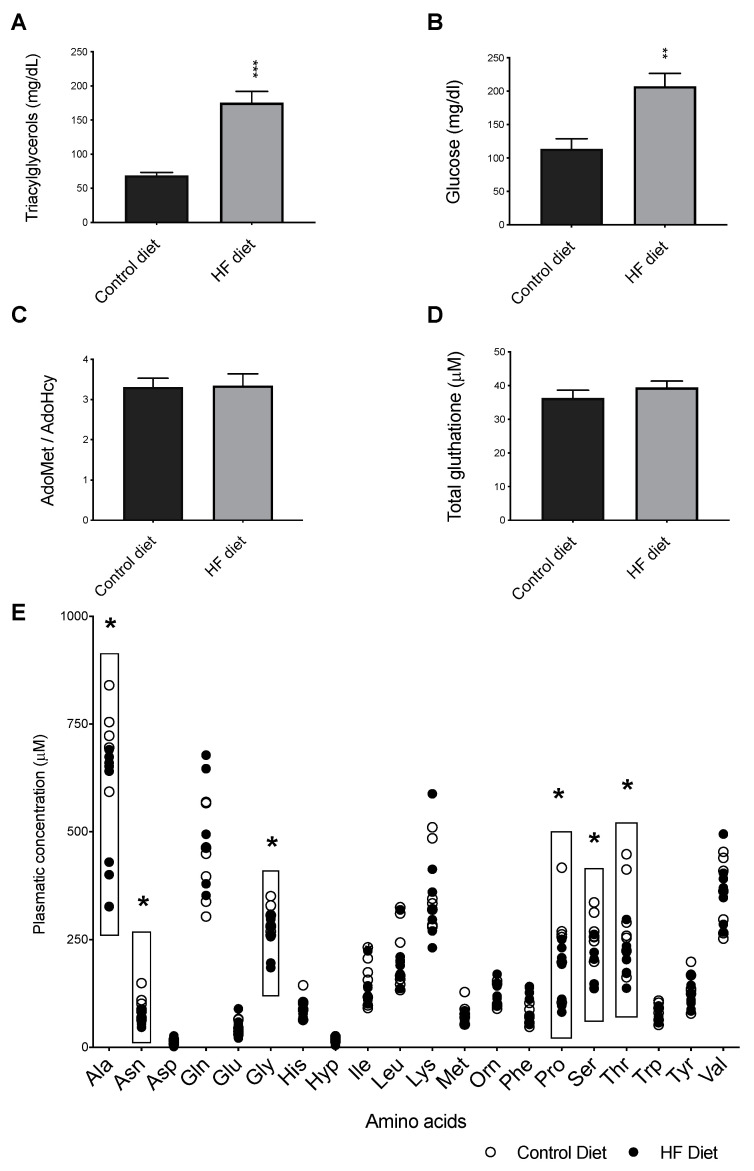
The effect of control and High-Fat (HF) diets on circulating concentrations of triacylglycerols (**A**), glucose (**B**), S-adenosylmethionine to S-adenosylhomocysteine ratio (AdoMet/AdoHcy) (**C**), total glutathione (**D**), and amino acids (**E**). (Ala, alanine; Gly, glycine; Val, valine; Leu, leucine; Ile, isoleucine; Thr, threonine; Ser, serine; Pro, proline; Asn, asparagine; Asp, aspartate; Met, methionine; Hyp, hydroxyproline; Glu, glutamate; Phe, phenylalanine; Gln, glutamine; Orn, ornithine, Lys, lysine; His, histidine; Tyr, tyrosine; Trp, tryptophan). Data shown are the mean ± SEM (n = 6–9 per group); * *p* < 0.05; ** *p* < 0.01; *** *p* < 0.001; HF versus control.

We next evaluated the effect of the HF diet on several biochemical parameters. The results showed that the mice fed a HF diet had significantly increased fasting plasma TAG (Figure 3A) and glucose levels (Figure 3B), thus confirming the major metabolic disturbances elicited by the HF diet previously described by others [31,41,42]. In fact, male *apoE*^−/−^ mice fed HF-diet are a widely used model of insulin resistance [43], a condition where aquaglyceroporins expression and function are affected [15,44,45].

#### 3.2.1. Systemic Methylation Index

The ratio of the metabolites, S-adenosylmethionine (AdoMet) to S-adenosylhomocysteine (AdoHcy), or AdoMet/AdoHcy measures the cell methylation potential [46,47,48]. AdoMet is the universal methyl donor to cellular methyltransferases, originating the methylated substrate and the metabolite AdoHcy. Importantly, AdoHcy may negatively regulate the activity of those same methyltransferases. Thus, a decreased AdoMet/AdoHcy ratio reflects a hypomethylating environment. In previous cell studies we have observed that a decrease in AdoMet/AdoHcy ratio downregulated AQP1 expression and promoted an atherogenic endothelial phenotype [14]. Yun et al. have reported that a diminished methylation index was present in wild type mice fed HF diets [49]. These observations led us to measure the systemic concentrations of AdoMet and AdoHcy in this study. Nevertheless, the results revealed similar plasmatic AdoMet/AdoHcy ratios in both groups of animals, thus showing that the systemic methylation index was not affected by the HF diet (Figure 3C) in *apoE*^−/−^ mice.

#### 3.2.2. Plasma Glutathione

The role of AQPs as facilitators of hydrogen peroxide membrane permeation, a major reactive oxygen species (ROS), has been recently reported [50]. Importantly, ROS build-up is a main pathophysiological mechanism favoring the establishment and progression of atherosclerosis [47]. Glutathione is a major cellular antioxidant that neutralizes hydrogen peroxide to water. Thus, we measured the systemic levels of total glutathione in our animals. The results, shown in Figure 3D, revealed similar plasma levels of glutathione in both groups of animals thereby suggesting that the bioavailability of this tripeptide is intact in the HF-mice. In support of this possibility, the plasma levels of three amino acids that form glutathione, i.e., glutamate (Figure 3E), glycine (Figure 3E), and cysteine (data not shown) did not differ between these two diets. We do acknowledge however that the measurement of the concentrations of both the free and reduced glutathione forms would be necessary to give a functional measure of this major antioxidant system.

#### 3.2.3. Plasma Amino Acids

Next, we evaluated the plasmatic levels of amino acids to visualize any changes driven by the HF diet that could suggest disturbed metabolic pathways to be further related with diet- induced effects on *AQPs* expression. In fact, recent developments have begun to shed light on associations between compromised cardiometabolic function and altered intermediary metabolism of amino acids [51]. In the present study, the plasma amino acid levels of the essential amino acids were not different between two groups (Figure 3E). Thus, the concentrations of the branched chain amino acids (BCAA), Tyr, and Phe, which were previously implicated in heart failure, a form of CVD [51,52,53], were similar in both the HF-fed and control groups. As shown in Table A1, both diets contained the same amount of casein, which was the only protein source. Because essential amino acids cannot be synthetized endogenously, this observation suggests that the metabolic pathways responsible for utilization and catabolism of essential amino acids are intact in the HF-fed animals. Among the remaining amino acids, significantly lower levels of alanine (Ala), asparagine (Asn), lysine (Lys), serine (Ser), and proline (Pro) were found in the plasma of the HF-fed mice compared to the control group (Figure 3E), suggesting that the HF diet resulted in a decreased protein turn-over state that is consistent with preferential utilization of the dietary fat as energy fuel [54].

#### 3.2.4. Systemic Inflammation

**Figure 4:**
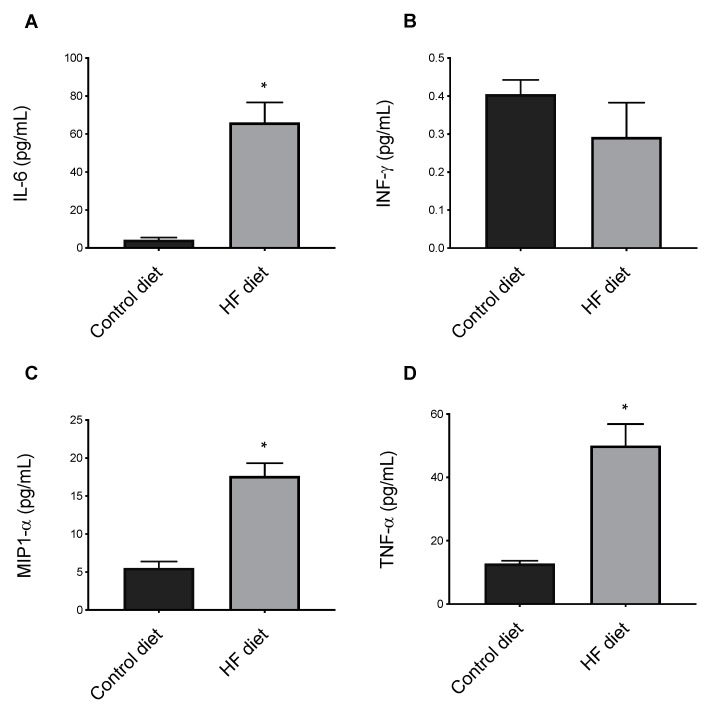
The effect of control and High-Fat (HF) diets on systemic inflammation. Interleukin 6, IL-6 (**A**); interferon gamma, INF-γ (**B**); macrophage inflammatory protein-1 alpha, MIP-1α/CCL3 (**C**); tumor necrosis factor alpha, TNF-α (**D**). Data shown are the mean ± SEM (n = 4 per group). * *p* < 0.05 HF versus control.

Finally, we determined the effect of the diets on plasma proinflammatory cytokines, including interferon gamma (IFN-γ), interleukin 6 (IL-6), macrophage inflammatory protein-1 alpha (MIP-1α/CCL3), and tumor necrosis factor alpha (TNF-α) [42,55,56]. IFN-γ is a proinflammatory mediator that is expressed in atherosclerotic lesions [57]. IL-6 is a pleiotropic cytokine with both pro- or antiatherogenic effects but which exacerbates atherosclerosis in murine species [58,59] MCP-1 is an inflammatory chemokine with a critical role in the initiation of atherosclerosis [60]. TNF-α is another major proatherogenic molecule that sustains the progression of the vascular lesions and atherosclerosis [42]. As expected, feeding mice with HF diet significantly elevated the systemic concentration of most cytokines, when compared to the control diet-fed mice (Figure 4). This observation is in agreement with the positive effect of dietary fat on systemic inflammation and with the well-established atherogenic effect of a HF diet in *apoE*^−/−^ mice [61,62]. In fact, inflammation is central to all stages of atherosclerosis establishment and progression.

### 3.3. Volume of Atherosclerosis

**Figure 5:**
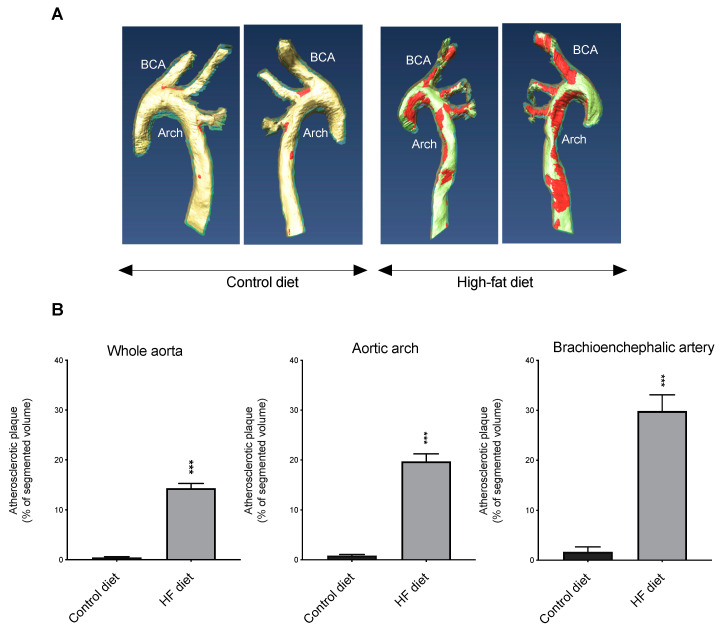
Differences in aortic atherosclerosis burden between mice fed the different diets. (**A**) Representative images (both sides of the same aorta are shown) of the visualization of the arteriosclerotic plaque (colored in red) using 14T-magnetic resonance imaging (MRI) in aortas from mice fed control or high-fat diets (BCA, brachiocephalic artery). (**B**) 14T-MRI volumetric assessment of the atherosclerotic plaque in different aorta segments. Data shown are the mean ± SEM (n = 8–10 per group). *** *p* < 0.001, HF versus control.

A method based on ex-vivo MR imaging was used to visualize and quantify the aortic volume of fatty plaque in HF-fed mice versus controls. The results revealed a profound difference of the atherosclerosis burden between the two groups of animals (Figure 5). Plaque volume throughout the whole aorta was significantly increased in the HF group compared to the control group; the atherosclerotic lesions predominantly distributed in the aortic arch and in the areas surrounding the branching points of the major arteries, which includes the brachiocephalic artery (BCA). As a result, the difference between HF-aortas and control aortas in these two highly susceptible regions, aortic arch and BCA, was even more pronounced than in the whole aortas. Interestingly, despite this massive difference of vascular lesions between HF-fed mice and controls both groups of animals presented similar plasma concentrations of the BCAA Val, Leu and Ile, and of Tyr and Phe (Figure 3), which were previously suggested to have a significant role in the pathogenesis of atherosclerosis and CVD [51,52,53]. The increased systemic inflammation observed in HF-fed mice, however, (Figure 4) is consistent with this augmented arteriosclerotic plaque burden observed in these mice. Taken together, these observations are in agreement with the well-established atherogenic effect of dietary fat [63]. Having induced a strong atherosclerotic phenotype with HF diet in *apoE*^−/−^ mice, when compared to controls, we determined the expression levels of the orthodox AQP isoforms, *AQP1* and *AQP5*, and of the aquaglyceroporins *AQP3*, *AQP7*, and *AQP9,* in both groups of mice.

### 3.4. Expression of Aquaporins in Liver and Adipose Tissues

The expression of *AQPs* with impact on metabolism and endothelial function (*AQP1*, *AQP3*, *AQP5*, *AQP7*, and *AQP9*) [14] was evaluated in three distinct metabolic tissues: liver, WAT and BAT. Liver is the organ responsible for the plasma glucose levels maintenance, thus, in a situation of negative energy balance or exercise, glucose is produced in the liver and released to the bloodstream [64]. When the body is in a state of positive energy balance and plasma glucose levels are high (as in the HF-fed mice), energy is stored in WAT in the triacylglycerol form, to be hydrolyzed in case of energy demands. BAT is a tissue that burns excess fat by thermogenesis [65].

**Figure 6:**
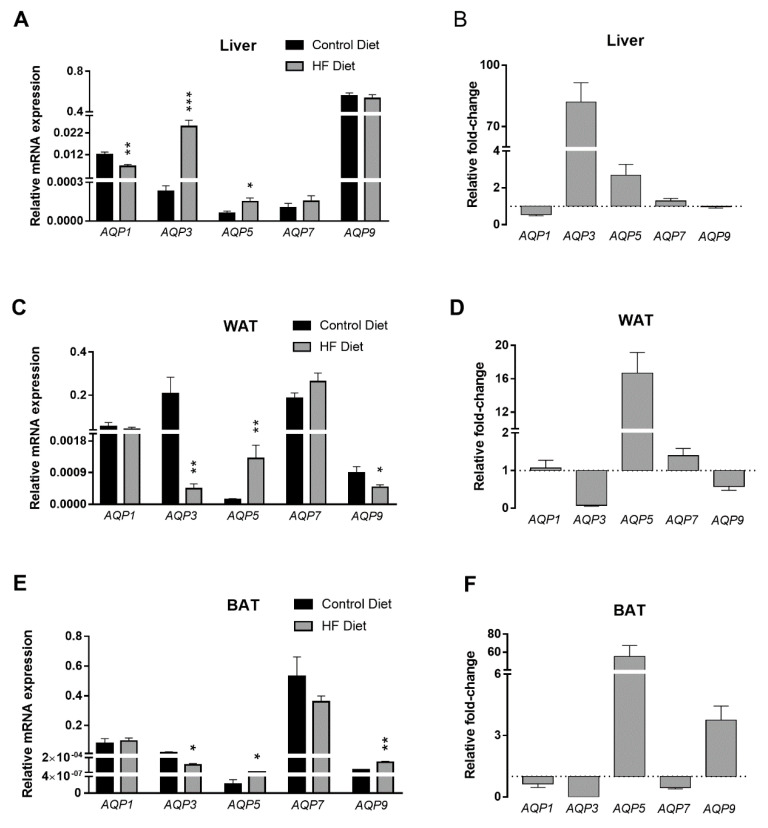
Aquaporins gene expression in *apoE*^−/−^ mice fed control or atherogenic High-Fat (HF) diets. *AQP1*, *AQP3*, *AQP5*, *AQP7* and *AQP9* expression in liver (**A**), WAT (**C**) and BAT (**E**) in mice fed control or HF diets and respective fold-change in HF-fed animals relative to controls [liver (**B**); WAT (**D**); and BAT (**F**)]. Results are mean ± SEM (n = 8–10 per group). * *p* < 0.05; ** *p* < 0.01; *** *p* < 0.001; all HF versus control.

All of the investigated *AQP* isoforms were detected in liver, WAT, and BAT, however, each in a tissue-specific profile (Figure 6A,C,E, black bars). In liver, and as previously described, *AQP9* was the most highly expressed isoform [18]. Moreover, *AQP1*, *AQP3*, *AQP7*, and *AQP5* were also detected in liver, although at lower levels than *AQP9* (Figure 6A). While AQP9 has been described as responsible for glycerol influx in hepatocytes, no specific function has been attributed to any other hepatic AQP isoform [66]. Concerning the impact of the HF diet on the hepatic *AQPs* expression levels, we observed that *AQP3* was the most affected isoform, with the HF-fed mice presenting levels that were around 80-fold more than controls. Nevertheless, since the overall expression level of *AQP3* was lower than the most abundant isoform *AQP9*, an increase in *AQP3* expression may not have significantly impacted the total glycerol efflux in hepatocytes. Interestingly, however, AQP3 is also a hydrogen peroxide channel [67], and thus its upregulation might represent an increase in oxidative stress in the tissue. This possibility is favored by the fact that the hepatic expression levels of another peroxiporin, *AQP5*, were also significantly upregulated by the atherogenic diet. In fact, overall oxidative stress is a major driver of the strong atherogenic phenotype observed in these HF-fed animals [46,48]. Additionally, the results showed that hepatic *AQP1* was significantly downregulated by the HF-diet whereas the levels of *AQP9* remained unaltered (Figure 6B). One limitation to our study is the fact that AQP9 immunolocalization was not performed, which would show whether it is correlated with gene expression or liver pathology. By histopathological analysis we could confirm the presence of hepatic fat accumulation in the HF-fed mice. Nevertheless, the lack of markers of hepatic fibrosis in the present study prevents us to further elucidate the relationship between hepatic *AQPs* expression and the development of atherosclerosis. However, a study in patients with morbid obesity did not find any relationship between AQP9 expression in the liver and the degree of hepatic steatosis or fibrosis [68]. Therefore, it seems that regulation of fatty liver deposits is not influenced by *AQP9* expression. Thus, it is unlikely that animals fed the atherogenic diet and with liver fat deposition show altered hepatic aquaglyceroporin expression profile.

Interestingly, the association of an inadequate AQP1 function with an atherogenic phenotype has been previously reported albeit in other contexts. For example, we observed that endothelial dysfunction under hypomethylating stress lessened *AQP1* expression in vitro [14], and others reported that the targeted deletion of the *AQP1* gene favored atherosclerosis progression in *apoE*^−/−^ mice [4]. In the present study, and as discussed above, the AdoMet/AdoHcy ratio was not affected by the HF-diet, so we can exclude a disturbed systemic methylation index as causing the observed HF-induced hepatic *AQP1* downregulation.

In WAT, we have detected abundant levels of *AQP1*, *AQP3* and *AQP7* mRNAs, while *AQP5* and *AQP9* were present in low amounts (Figure 6C) [16,66]. A strong induction of *AQP5* expression of around 15-fold was observed in mice fed HF diet. As aforementioned, *AQP5* is a peroxiporin whose expression has been related with oxidative stress in rodents [69] and humans [13]. We have previously reported dysregulation of endothelial AQP5 expression associated with endothelial dysfunction [14], suggesting AQP5 is closely related to a dysbalanced redox state that favors the atherosclerosis progression. In addition, our present results show that *AQP3* and *AQP9* expression is impaired in WAT from mice fed HF diet (Figure 6D). Knowing that AQP3 and AQP9 are the most abundant aquaglyceroporins in mice adipose membranes [26], their downregulation might represent a cellular strategy to avoid excess glycerol efflux to be used in liver.

BAT presented high levels of *AQP7* gene expression, the most representative isoform in this tissue, and low levels of *AQP1*, *AQP3*, *AQP5*, and *AQP9* mRNAs (Figure 6E). In the BAT of HF-fed mice, *AQP3* was downregulated, *AQP9* and *AQP5* were increased around 4- and 60-fold, respectively, while *AQP1* and *AQP7* expression was unaltered (Figure 6F). The downregulation of *AQP3* in parallel with the dramatic upregulation of *AQP5*, suggests an impairment of glycerol movements coupled to increased sensitivity to oxidative stress, similar to the observed in WAT.

The *AQPs* expression pattern in the three analyzed tissues revealed tissue-specific differences, inherent to their predicted metabolic role. However, *AQP5* expression was consistently induced by HF diet across WAT, BAT, and liver. Since AQP5 facilitates hydrogen peroxide permeation in rodents and humans, its upregulation suggests the presence of an unbalanced redox state [13,69]. Here, we have detected similar plasma levels of total glutathione, the major intracellular antioxidant that neutralizes hydrogen peroxide, in both groups of animals, thereby suggesting that the bioavailability of glutathione was intact in the HF-fed mice. Nevertheless, this similar glutathione bioavailability does not translate into a similar antioxidant capacity and could only be evaluated by determining the concentration of the reduced and oxidized glutathione forms. AQP5 dysfunction has been associated with a vast array of phenotypes, and evidence suggests that AQP5 upregulation promotes tumor cell proliferation [70,71]. Studies correlating AQP5 to obesity are scarce; however, a link between hypothalamus *AQP5* and adipocyte gene expression was reported, indicating a possible regulatory coordination [72]. Interestingly, AQP5 is crucial for mice adipocyte differentiation [73] and *AQP5*-KO mice have lower body weight than controls [74]. Altogether, these data suggest that the increased *AQP5* expression observed in WAT may indicate an increase in adipocyte differentiation to accommodate the excess fat in HF diet.

## 4. Conclusions

In conclusion, the present study contributes to a better characterization of the well-established *apoE*^−/−^ mouse model by reporting that the pattern of *AQPs* expression in these mice is disturbed, in a tissue-specific manner, by an atherogenic HF diet. The present report suggests a novel relation between diet, AQP5, and atherosclerosis that warrants further investigation and may ultimately open the door to the development of effective new treatments for CVD.

## Data Availability

Not applicable.

## Appendix A

**Table A1:** Macronutrient composition of the experimental diets.

	Control Diet		High Fat Diet	
	g	*Kcal*	g	*Kcal*
Casein	180	720	184	736
L-Cysteine	3	12	3	12
Corn starch	431	1725	217	868
Maltodextrin 10	155	620	93	372
Sucrose	100	400	102	408
Cellulose	35	0	35	0
Cocoa butter	0	0	155	1395
Primex	25	225	0	0
Corn oil	25	225	25	225
Cholesterol	0	0	11	0
